# Special Issue: Immune-Mediated Neurological Disorders

**DOI:** 10.3390/biomedicines11123214

**Published:** 2023-12-04

**Authors:** Luca Marsili, Carlo Colosimo

**Affiliations:** 1 Gardner Family Center for Parkinson’s Disease and Movement Disorders, Department of Neurology, University of Cincinnati, Cincinnati, OH 45219, USA; 2 Department of Neurology, Santa Maria University Hospital, 05100 Terni, Italy; c.colosimo@aospterni.it

For a long time, the immune system has been considered responsible for only a minority of neurological conditions involving the central and peripheral nervous system (CNS, PNS), respectively, namely multiple sclerosis and myasthenia gravis (with myastheniform syndromes). More recently, with recent advances in biology, immunology, and neurosciences, it has become evident that the immune system plays an essential role in affecting the interaction of our organism with the external world, including infectious diseases, toxic agents, drugs, and other environmental substances. The immune system should now be considered as the leading player in multiple conditions that may be observed in neurology clinical practice and that general neurologists should know about [1]. Also, it is crucial to know the possible effects of new immune-modulating treatments, frequently used in oncology, that may contribute to some of these conditions [2]. Finally, the role of the immune system in neurodegeneration is still largely unknown, but immune signals could be possibly used as biomarkers of disease progression once this role has been clarified [3]. This Special Issue was designed to provide an overview of the immune system’s role in several neurological diseases, even those that are not classically considered immune-mediated. All the nine papers initially submitted to the journal by international researchers who are experts in the field were considered suitable for publication after a thorough peer review process. These included one original research article, six reviews, and two case reports/series. The following is a short summary of the main results of each of these manuscripts.

Vlad and colleagues (Contribution 1) have revised the main pathophysiological, clinical, and treatment perspectives of stiff-person spectrum disorders (SPSDs). SPSDs represent autoimmune movement disorders characterized by the combination of stiffness, spasms and hyperekplexia. SPSDs, even though relatively rare, are a “not-to-miss” diagnosis because of their significant disease burden and possible full recovery if promptly recognized and treated. After having been considered an orphan disease for decades, major advances in the understanding of the disease spectrum have been made after the identification of multiple associated autoantibodies. SPSDs may manifest with multiple different symptoms, beyond the classic core motor symptoms, thus rendering the diagnosis more difficult. Finally, new immunomodulatory and symptomatic therapies are now available for better management of these conditions.

In their case series, Neațu and coworkers (Contribution 2) have explored autoimmune encephalitis (AE) disorders, a complex and multifaceted pathology that involves immune-mediated CSF inflammation. A wide range of causes, mechanisms, and clinical presentations are associated with AE. In fact, the clinical presentation of AE is broad and can mimic other neurological conditions, thus making it a challenging diagnosis for clinicians. This diverse clinical presentation can overlap with other conditions, making it crucial for clinicians to maintain a high level of suspicion for AE when evaluating these patients. Due to the variable nature of AE, there is no definitive diagnostic test that can confirm the diagnosis, but multiple clinical, neuroimaging, and laboratory clues have been proposed. In addition, patients’ treatment plans should be tailored to the specific clinical presentation, underlying cause, and immune response.

Marsili et al. (Contribution 3) focused on the paraneoplastic neurological syndromes (PNSs), namely any symptomatic and non-metastatic neurological manifestations associated with a given cancer. PNSs may be associated with antibodies against intracellular antigens, known as “high-risk” antibodies, and in this case, show frequent association with underlying cancer. PNSs associated with antibodies against neural surface antigens, known as “intermediate- or low-risk” antibodies, are less frequently associated with cancer. Clinicians should have a high index of suspicion with acute/subacute encephalopathies to achieve prompt diagnosis and treatment. PNSs of the CNS exhibit a range of overlapping “high-risk” clinical syndromes, including, but not limited to, latent and overt rapidly progressive cerebellar syndrome, opsoclonus–myoclonus–ataxia syndrome, paraneoplastic (and limbic) encephalitis/encephalomyelitis, and stiff-person spectrum disorders. Moreover, these clinical presentations may also arise as an adverse event of the recent anti-cancer treatments belonging to the categories of the immune-checkpoint inhibitors and CAR T-cell therapies. These therapies cause a boosting of the immune system against cancer cells, and the related clinical manifestation arises as a consequence of these excessive immune effects. The field of PNSs of the CNS is constantly expanding with newly discovered antibodies and syndromes every year. Standardized diagnostic criteria and disease biomarkers are fundamental to quickly recognize PNSs to allow prompt treatment initiation, thus improving the long-term outcome of these conditions.

In their case report, Sorokina and colleagues (Contribution 4) described a case with a novel mutation in the SAMHD1 gene, causing a variant of Aicardi–Gouteres syndrome, type 5, manifesting with dermatomyositis and infantile cerebral palsy in a young girl. They showed how timely immune treatment can help avoid the development/progression of end-organ damage, including severe neurological complications and early death in these patients.

Boura and coworkers (Contribution 5) have examined in detail the immune-related effects of severe acute respiratory syndrome coronavirus-2 (SARS-CoV-2), which causes coronavirus disease 2019 (COVID-19), in patients with Parkinson’s disease (PD). Three years after the onset of the pandemic, it is of great interest to see how the community of patients with PD has been impacted by COVID-19. Since the pandemic started, many authors have suggested a possible role of COVID-19 as a contributing cause for PD; however, it is more likely that this viral infection may act as a trigger in patients with prodromal and/or asymptomatic PD. In any case, similar to other systemic infections, the worsening of PD symptoms secondary to COVID-19, either transient or persistent (long COVID), has been demonstrated. Also, COVID-19-related mortality in PD patients seems to be increased compared to the general population. These observations could be attributed to direct or indirect damage from SARS-CoV-2 in the CNS or could result from general infection-related parameters and the consequences of the COVID-19 pandemic. The onset of PD following SARS-CoV-2 infection could be either closely (post-infectious) or remotely (para-infectious) related, and this link still remains hypothetical. The pathophysiological substrate of these phenomena remains unclear; however, pathology studies have suggested various COVID-19-induced degenerative changes with potential associations with PD.

Monroy et al. (Contribution 6) discussed the immune and inflammatory pathways linked to neurodegeneration. Immunization with neural-derived peptides (INDPs) is a relatively novel treatment consisting of inoculation of neural-derived peptides obtained from the CNS. This therapy aims to boost protective autoimmunity, thus producing a healing environment and neuroregeneration instead of causing neuronal death. INDP has shown promising findings in studies performed either in vitro, in vivo in animal models, or even in some pre-clinical trials of different neurodegenerative diseases. These immune-mediated therapeutic avenues should be further explored in neurodegeneration to see if they may represent a valid and safe rescue approach.

In his review (Contribution 7), Pandey explored the mechanisms of neuroinflammation in lysosomal storage diseases, a group of genetic disorders caused by accumulation of toxic substances in the lysosome. This accumulation of cellular materials causes the activation of immune cells, leading to neuroinflammation and neurodegeneration. The resulting inflammatory environment leads to the generation of pro-inflammatory cytokines, chemokines, growth factors, and several components of complement cascades, which contribute to the progressive neurodegeneration seen in these diseases. By understanding the mechanisms behind these diseases, potential biomarkers, and therapeutic targets for them could be designed in the near future.

Giannoccaro and colleagues (Contribution 8) investigated the relationship between AE and neurodegeneration. Interestingly, antibodies against neuronal proteins, such as those directed against NMDAR, have been found in patients with neurogenerative disorders, but their role is still unclear. They might be simple bystanders or modify the disease course. However, specific antibodies to neuronal surface antigens have been associated with neurodegenerative features. A paradigm of the neurodegenerative role of autoantibodies is IgLON5 antibody-related encephalitis (cognitive impairment, movement disorders, and sleep disorders), a unique model of the relationship between antibodies and neurodegeneration. The detailed physiological and pathological study of these antibodies will shed light on mechanisms of neurodegeneration. The underlying mechanisms of movement disorders caused by IGLON5-related diseases have been also explored in the original article by Gao et al. (Contribution 9). They purified anti-IGLON5 antibodies from the serum of an affected patient and then passively transferred them into the substantia nigra pars compacta (SNc) of a mouse model. The effects of anti-IgLON5 antibodies on dopaminergic neurons in the SNc and neurodegeneration were examined using immunohistochemistry. They noticed that purified serum IgG from a patient with anti-IgLON5 antibodies can cause long-term movement disorder in mice, mostly related to an impairment in dopaminergic pathway. They also noticed an increase in p-Tau, which caused long-term neurodegenerative changes induced by the anti-IgLON5 antibody.

All the articles published in the present Special Issue underline the crucial role of the immune system in triggering or amplifying both acute and chronic neurologic disorders, even those not classically considered immune-related. This aspect suggests that a better knowledge of immune mechanisms is required to understand the pathophysiology of multiple neurologic diseases including neurodegeneration, in some cases. Immune therapies may be used to treat not only acute but also chronic neurologic illnesses. Also, anti-cancer immune-boosting therapies may have autoimmune neurological syndromes as adverse events. Clinicians should be aware of the management of these anti-cancer treatment adverse events and be able to temporarily hold off these treatments until patients recover, but timely re-start of these powerful treatments should be considered [4].

In conclusion, the Editors wish to thank all the authors, the reviewers, and the Editorial Board Members for contributing to this Special Issue. We hope that this Special Issue might inspire future and novel research approaches relating to the role of the immune system in several neurological disorders.
